# CT45A1 promotes the metastasis of osteosarcoma cells in vitro and in vivo through β-catenin

**DOI:** 10.1038/s41419-021-03935-x

**Published:** 2021-06-25

**Authors:** Mingxin Wen, Hui Ren, Shouqiang Zhang, Tao Li, Jiefeng Zhang, Peng Ren

**Affiliations:** 1 Department of Anatomy, Shandong University School of Medicine, Jinan, Shandong PR China; 2 Department of thoracic surgery, Central Hospital of Xinwen Mining Group Company, Xintai, Shandong PR China; 3 Department of Traumatology, The Second Hospital of Shandong University, Jina, PR China; 4 Trauma department of orthopedics, Shandong University School of Medicine, Jinan, Shandong PR China; 5 Department of Traumatology, Taian City Central Hospital, Taian, PR China; 6 Trauma department of orthopedics, The Second Hospital of Shandong University, Jina, PR China

**Keywords:** Epithelial-mesenchymal transition, Sarcoma

## Abstract

Increased expression of cancer/testis antigens (CTAs) is reported in various tumors. However, the unique role of CTAs in tumor genesis has not yet been verified. Here, we first report the functional role of CT45A1 in the carcinogenesis of osteosarcoma. RNA sequencing and immunohistochemistry confirmed that elevated expression of CT45A1 was detected in osteosarcoma, especially in metastatic tissues of osteosarcoma. Furthermore, osteosarcoma patients with poorer prognosis showed high expression of CT45A1. In cell tests, CT45A1 overexpression was shown to strengthen the proliferation, migration, and invasion abilities of osteosarcoma cells, while silencing CT45A1 markedly elicited the opposite effects in these tests by disrupting the activation of β-catenin. In summary, we identify a novel role of CT45A1 in osteosarcoma. Furthermore, our results suggested that CT45A1 may contribute to the development of osteosarcoma and could be a possible therapeutic target for osteosarcoma patients.

## Introduction

Osteosarcoma is a rare mesenchymal tumor but is the most reported malignant bone tumor in persons aged less than 25 years old [1], with the highest morbidity in people aged from 10 to 20 years old [2]. Induced mesenchymal cells and bone destruction are the characteristics of osteosarcoma [3]. In recent years, the diagnosis and therapy of osteosarcoma have improved; however, the prognosis of some osteosarcoma patients remains unsatisfactory because of the high rate of metastasis to the lungs [4, 5]. Therefore, novel markers for the metastasis of osteosarcoma are essential for reducing the mortality of osteosarcoma patients. This research summarizes the biological behavior and treatment strategy of osteosarcoma. Considering, the poor prognosis of osteosarcoma patients, we attempted to find a novel signaling pathway that participates in the progression or metastasis of osteosarcoma. Based on our research, we revealed a novel gene in the metastasis of osteosarcoma and suggested a possible therapeutic target for osteosarcoma patients.

According to reports, epithelial–mesenchymal transition (EMT) can be induced by multiple signaling pathways in tumor invasion and metastasis [6–10], which are key biological changes of tumors. Cancer cells with EMT more easily gain invasion and migration abilities and have a greater chance of survival after chemotherapeutic treatment [11, 12]. During EMT, obvious changes are detected both at the DNA and protein levels, and cell morphological changes are seen following EMT. As reported, loss of epithelial (E)-cadherin on the membrane is the critical step of EMT that disrupts cellular adhesion and allows the cells to separate from solid tumors and obtain the ability to cross the basement membrane [13]. Particularly, cancer cells with EMT show a greater ability to metastasize.

Multiple CTAs are reported to be abnormally expressed in tumors, and tumor patients with aberrant CTAs commonly show poorer prognosis [14–17]. Previously, various CTAs have been reported and the aberrant expression of CT45, a type of CTAs, is found in many types of tumors [18]. Six splicing forms of CT45 have been detected, named CT45A1-6 which are in a tandem arrangement at chromosome Xq26.3 [19]. As shown previously, the *CT45* gene is commonly expressed in testis, but aberrant *CT45* genes are also found in different tumor tissues such as myeloma [20], lung cancer [21], colon cancer [22], and breast cancer (BC) tissues [23]. *CT45* genes are associated with poorer prognosis of these cancer patients. Moreover, the expression level of *CT45* in BC is associated with the more radical biological behavior of BC cells, which suggests us that *CT45* may be a novel therapeutic target for BC patients [24]. These data suggested us that CT45 may act as an oncogene in tumor genesis and may be a potential marker in diagnosis and prognosis of tumors.

Here, we first report the functional role of CT45A1, a member of the CT45 gene family, in the carcinogenesis of osteosarcoma. Bioinformatics analysis showed us that CT45A1 was associated with the prognosis of osteosarcoma. Cell tests revealed the positive role of CT45A1 in the proliferation, viability, and metastasis of osteosarcoma cells. We also revealed that CT45A1 promoted the activation of β-catenin and induced metastasis of osteosarcoma.

## Materials and methods

### Cancer tissues and cell lines

We obtained approval from the ethics committee of the Second Hospital of Shandong University. A total of 20 pairs of osteosarcoma tissues and adjacent tissues were restored in 4% paraformaldehyde or liquid nitrogen and the related information of the tumor stage was obtained from the pathological department of the Second Hospital of Shandong University. The osteosarcoma cell lines U2OS, MG63, and HOS were purchased from the American Type Culture Collection (ATCC) and cultured in the medium indicated by the ATCC introduction supplemented with 10% fetal bovine serum (Gibco) and 1% streptomycin/penicillin under an atmosphere comprising 5% CO_2_ at 37 °C.

### Retroviral transduction

Retroviruses of pBabe, pBabe-CT45A1, pSuper, pSuper-shCT45A1 #1 or #2 were prepared by Phoenix packaging cells. Moreover U2OS, MG63, and HOS cells were transfected with different viral supernatants, and next, 4 μg/ml polybrene was added to the samples (Merck, TR-1003). After 48 h, 2 μg/ml puromycin (Tocris 4089) was used to select positive cells. The shRNA sequences of *CT45A1* at 3′-UTR were:

#1CCGGCCAGCCAATTGGATTCTCAGACTCGAGTCTGAGAATCCAATTGGCTGGTTTTTTG; #2CCGGACGAGAAATTAATGCTGATATCTCGAGATATCAGCATTAATTTCTCGTTTTTTG.

### Quantitative reverse transcription-polymerase chain reaction (qRT-PCR)

The RNA of tissues frozen in liquid nitrogen or cells were prepared by TRIzol reagent (Invitrogen). Then, cDNA was generated by a First Strand cDNA Synthesis Kit (Invitrogen). The qPCR analysis was carried out in a PRISM7900HT system (ABI) using SYBR Green PCR Mix (Invitrogen). The brief steps of the qPCR reaction were as follows: denaturation at 95 °C for 15 min, 40 cycles at 94 °C for 10 s, and 58 °C, 1 min. The results were analyzed by SPSS:

*GAPDH* forward- CCCCAAAGCAAAGAAACATACC,

reverse- TCACTTCCAGTTATTAGCAACAAT;

*CT45A1* forward- GCTGTCTCTCCTCCAGCAAGGAAAC,

reverse- GACTGCAGTAGGTCCTTGCACTCCT;

*β-catenin* forward- TAGAAGTCTGAACACTCGTT,

reverse- AATTCCTCTGATTGTTACCATT;

*c-Myc* forward- CAGTTCCGGAGGTACTTGGA,

reverse- TGAGCAAGCTTTGCTTTCAG;

*CyclinD1* forward- GGAATTGATGCGTGATGT,

reverse- ACCAGGTGCTGTGGAGTA.

### Transwell assay and matrigel assay

Osteosarcoma cells were seeded into upper chambers (BD transwell chambers), with or without the basement matrix. For the transwell assay, 2 × 10^4^ cells were seeded, and 5 × 10^4^ cells were seeded in a matrigel assay [25].

### Antibodies and agents

The antibodies were used at the following concentration: CT45A1 (1:1000, LS-C203655), E-cadherin (1:1000, CST3195), vimentin (1:1000, CST9856), α-SMA (1:1000, CST19245), c-Myc (1:1000, CST18583), CyclinD1 (1:1000, CST55506), Myc (1:1000, CST2276), Flag (1:1000, CST14793), β-catenin (1:1000, CST8480) and GAPDH (1:1000, CST5174).

XAV-999 was bought from MedChemExpress (CAS No.: 284028-89-3); ICG-001 was bought from APExBIO (Catalog No. A8217), both were used according to the instructions.

### Immunohistochemistry (IHC) analysis

Following the deparaffinization of the paraffin-embedded osteosarcoma tissue sections, the expression of CT45A1 in the osteosarcoma tissues was detected using a DAB HRP Kit (Beyotime, P0203). Nonspecific binding was blocked by incubating the sections with normal goat-serum for 30 min at room temperature. Endogenous peroxidase activity was quenched by incubating sections in 3% H_2_O_2_ in PBS for 20 min. Sections were then incubated with anti-CT45A1 monoclonal antibody at 4 °C overnight and washed with PBS (3 × 5 min) before incubating with secondary antibody for 30 min. Slides were washed again (3 × 5 min) with PBS before incubating with the ABC solution. The reaction color was developed by incubating sections with 3,3′-diaminobenzidine reagent. The slides were washed with water and counterstained with hematoxylin. The sections were then dehydrated and mounted with Permount permanent mounting media (Fisher Scientific, Fair Lawn, NJ). All slides were observed under Nikon E400 Light Microscope and representative photographs were taken.

### MTT assay

The indicated cells (1500 cells per sample) were seeded onto 96-well plates. Four hours after the MTT addition, the supernatant was removed, and DMSO was added. Then, the absorbance of the samples at 570 nm was measured by a microplate reader.

#### Tumor formation and metastasis in vivo

Nude mice were purchased from Shanghai Slac Laboratory Animal Co. Ltd. and maintained in microisolator cages. All animals were used in accordance with institutional guidelines and the current experiments were approved by the Use Committee for Animal Care.

For tumor formation in vivo, according to completely randomized principle, 10 mice were divided in to two groups and cells were resuspended in PBS at a concentration of 5 × 10^7^ cells/ml. Cell suspension (0.1 ml) was injected subcutaneously at the axillary. And measure the volume of tumors every 2 days.

For metastasis assays, according to completely randomized principle, 20 mice were divided in to four groups and U2OS and HOS with overexpressed CT45A1 and control cells were resuspended in PBS at a concentration of 3 × 10^7^ cells/ml. Cell suspension (0.1 ml) was injected into tail veins of nude mice. The mice were killed by 4% chloral hydrate 60 days after inoculation and the lungs were fixed and checked by hematoxylin and eosin (HE) stain.

### Statistical analysis

All data in this paper were expressed as the means ± SDs. The Kaplan–Meier method was used in survival analysis. Data were statistically compared using one-way ANOVA. *P* < 0.05 was considered statistically significant.

## Results

### Overexpression of CT45A1 is positively associated with metastasis and poor prognosis of osteosarcoma

**Fig. 1:**
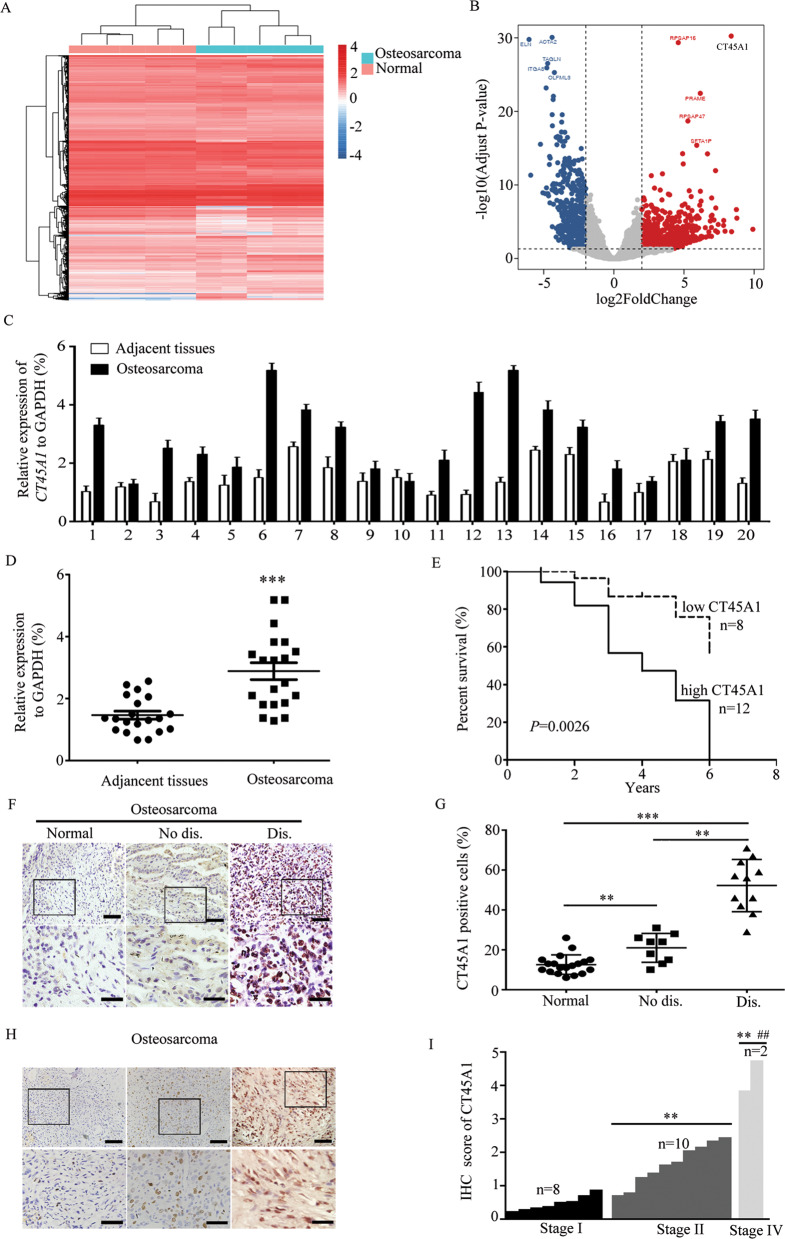
CT45A1 was related to the prognosis of osteosarcoma. **A** mRNA-seq between osteosarcoma tissues and adjacent tissues. **B** Bioinformatics analysis to get the under-/over-expressed genes in osteosarcoma. **C** Quantification of *CT45A1* expression in osteosarcoma tissues and relevant adjacent tissues. **D** Statistical analysis of *CT45A1* levels in osteosarcoma tissues and relevant adjacent tissues, ***P < 0.001, compared to adjacent tissues. **E** Survival analysis of patients with osteosarcoma in a dataset collected from the Second Hospital of Shandong University. **F** Immunohistochemical analysis of CT45A1 in osteosarcoma. Normal, Adjacent tissues; No dis., cancer tissues without lung metastasis; Dis., cancer tissues with lung metastasis. Scale bars: up 200 μm, down 150 μm. **G** Score analysis of CT45A1 expressions in (**F**), **P < 0.01, ***P < 0.001. **H** Immunohistochemical analysis of CT45A1 in tissue chip of osteosarcoma. Scale bars: up 200 μm, down 150 μm. **I** Score analysis of CT45A1 expressions in (**H**), **P < 0.01, compared to stage I; ^##^*P* < 0.01, compared to stage II. The error bars indicated the means ± SDs.

In this paper, we first tried to find possible genes that were related with the progression of osteosarcoma. Then, we analyzed the gene data of five pairs of osteosarcoma tissues and adjacent tissues to explore the possible genes that associated with the progression of osteosarcoma by RNA sequencing. Interestingly, we observed the upregulated gene *CT45A1* in gene data with high *P* value (Fig. 1A, B). When we examined the CT45A1 expression in normal or osteosarcoma tissues by qRT-PCR we found that CT45A1 was overexpressed in osteosarcoma tissues, compared to the adjacent tissues. (Fig. 1C, D). Moreover, bioinformatics analysis of osteosarcoma patients was conducted to further confirm this relationship. Whereas patients with poor prognosis showed high expression levels of CT45A1, patients with lower expression of CT45A1 had prolonged survival, thereby suggesting that CT45A1 is a prognostic marker of osteosarcomas (Fig. 1E). And in immunohistochemical staining, results were consistent with that in qRT-PCR (Fig. 1F). And surprisingly, we found that the more aberrant expression of CT45A1 was detected in osteosarcoma patients with distant metastasis than those without distant metastasis (Fig. 1F, G) as well as that higher expression of CT45A1 was also detected in osteosarcoma patients with more aggressive clinical pathological grade which suggested us that CT45A1 was related with metastasis of osteosarcoma. These results showed us that CT45A1 was significantly correlated with the metastasis and prognosis of osteosarcoma and future research was needed.

### CT45A1 promotes the proliferation of osteosarcoma cells

**Fig. 2:**
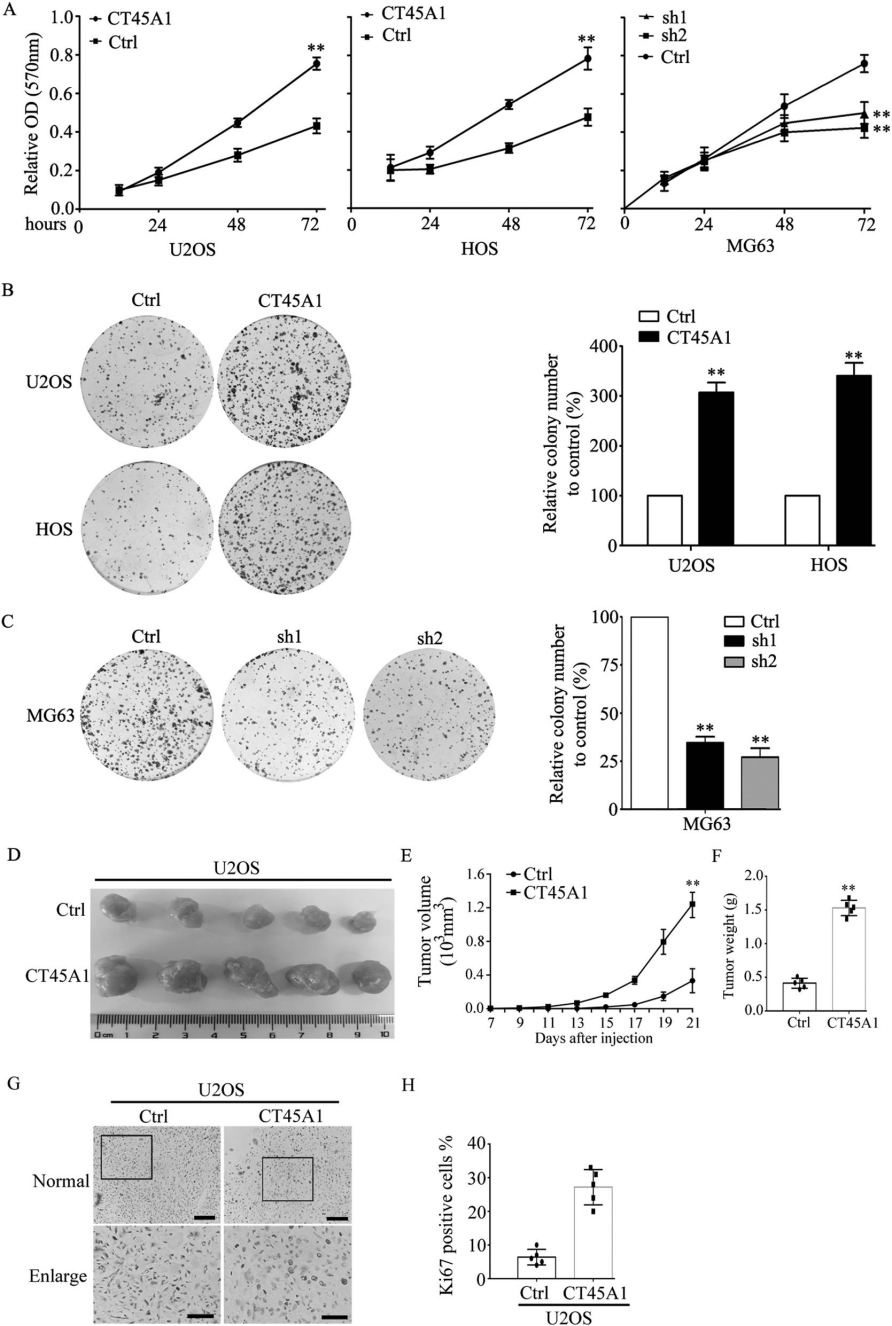
CT45A1 promoted the proliferation and viability of osteosarcoma cells. **A** MTT assay of the osteosarcoma cells with CT45A1 overexpression or silencing. **B** Colony formation of osteosarcoma cells with CT45A1 overexpression. **C** Colony formation of osteosarcoma cells with CT45A1 silencing. **D** Image of tumors in tumor formation in vivo. **E** Tumor volume analysis of osteosarcoma cells with CT45A1 overexpression. **F** Tumor weight analysis of osteosarcoma cells with CT45A1 overexpression. **G** Immunohistochemical analysis of Ki-67 in tumors with CT45A1 overexpression. Scale bars: 150 μm. **H** Score analysis of Ki-67 expressions in (**G**). **P < 0.01, compared to the control. The error bars indicated the means ± SDs. Ctrl indicates the control; sh1 and sh2 indicate MG63 cells with two different shRNAs of *CT45A1*.

To confirm the role of CT45A1 in osteosarcoma further, we established osteosarcoma cells with CT45A1 overexpression or silencing based on the basal expression of CT45A1 in osteosarcoma cell lines (Supplemental Fig. 1A, B), and the indicated levels of CT45A1 were detected by western blotting assay (Supplemental Fig. 1C, E) and qRT-PCR (Supplemental Fig. 1D, F). Then, the proliferative rate of osteosarcoma cells with different levels of CT45A1 was examined. The results showed that CT45A1 overexpression significantly promoted the proliferation of U2OS and HOS cells, while CT45A1 silencing inhibited the proliferative rate of MG63 cells (Fig. 2A). Moreover, in colony formation assay, CT45A1 overexpression also significantly promoted the proliferation of U2OS and HOS cells (Fig. 2B) while CT45A1 silencing suppressed the proliferation of MG63 cells (Fig. 2C). These data revealed that CT45A1 could promote the proliferation of osteosarcoma cells in vitro. Next, we detected the proliferated rate of osteosarcoma cells in vivo. Tumor formation assay in vivo revealed that tumors with overexpressed CT45A1 growth more rapidly and bigger that cells in control group (Fig. 2D–F) which was further supported by the higher expression of Ki-67 in tumors with overexpressed CT45A1 by IHC (Fig. 2G, H). These results above confirmed the role of CT45A1 in promoting the proliferation of osteosarcoma cells suggesting that CT45A1 is positively correlated with the growth of osteosarcoma cells.

### CT45A1 promotes the metastasis of osteosarcoma cells through upregulating EMT-related genes

**Fig. 3:**
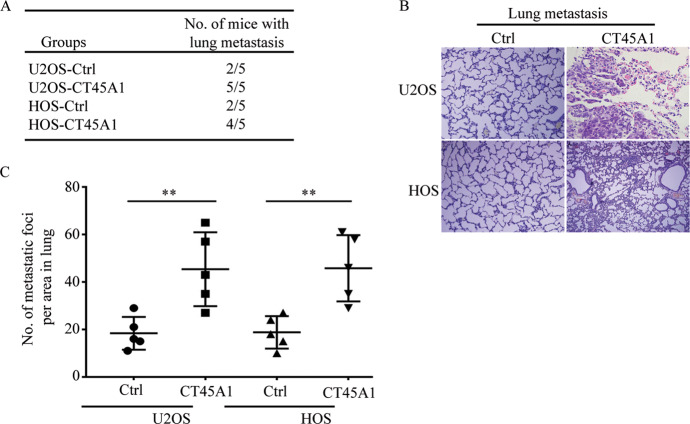
CT45A1 promotes the metastasis of osteosarcoma cells in vivo. **A** The number of mice injected with U2OS and HOS cells showing that CT45A1 overexpression exhibited distant metastasis. **B** Hematoxylin and eosin staining for metastatic foci in lung injected with U2OS and HOS cells with CT45A1 overexpression. Scale bars: 200 μm. **C** Statistic analysis of the number of metastatic foci in the lungs injected with U2OS and HOS cells with CT45A1 overexpression. **P < 0.01, compared to the control.

**Fig. 4:**
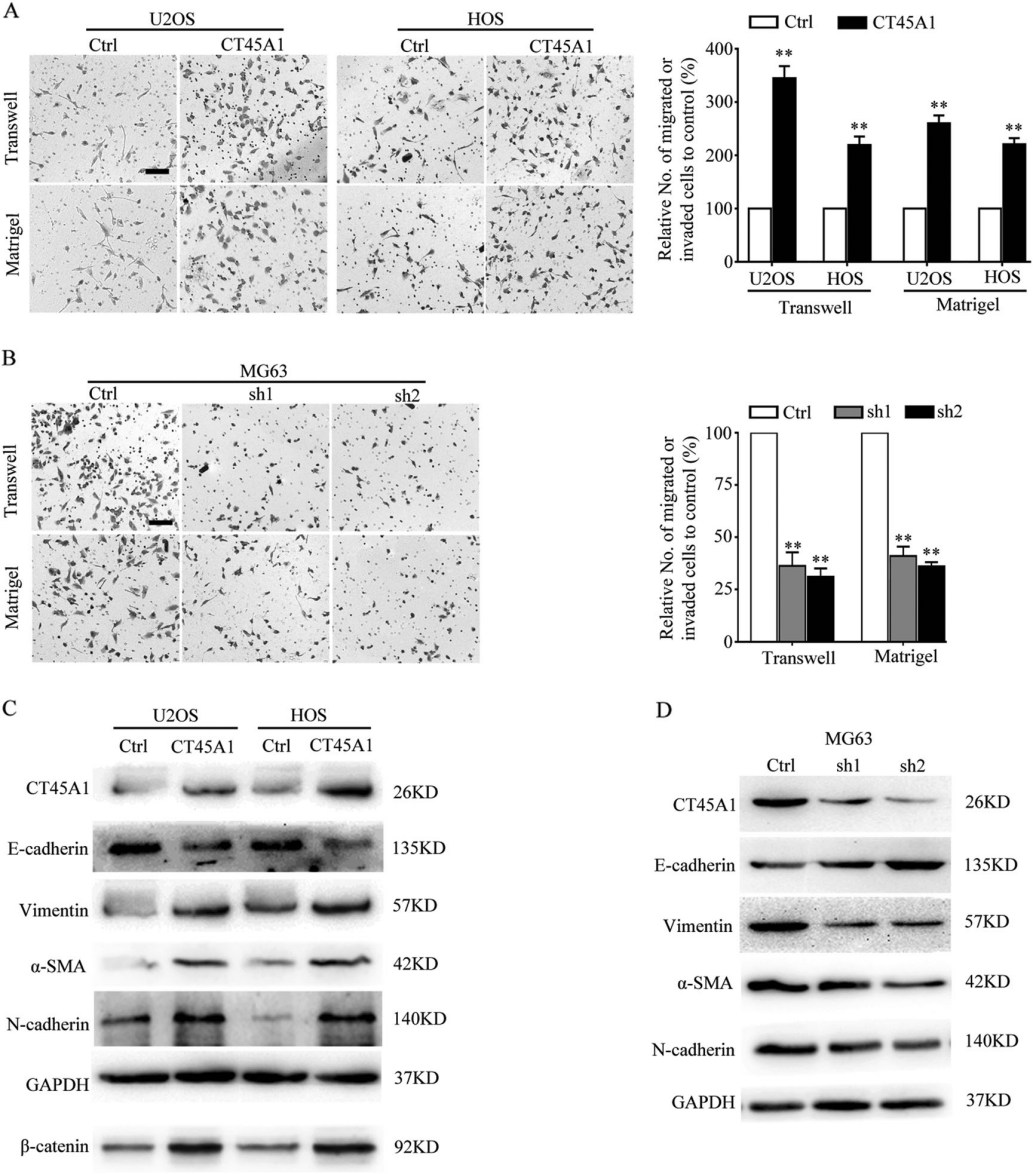
CT45A1 promotes the metastasis of osteosarcoma cells in vitro by inducing EMT. **A** Transwell and martrigel assay of osteosarcoma cells with CT45A1 overexpression. **B** Transwell and martrigel assay of osteosarcoma cells with CT45A1 silencing. **C** Western blotting for the quantification of EMT markers in osteosarcoma cell lines with CT45A1 overexpression. **D** Western blotting of EMT markers in osteosarcoma cell lines with CT45A1 silencing. Scale bars: 100 μm (**A**, **B**). **P < 0.01, compared to the control. The error bars indicate the means ± SDs.

Aberrant proliferation, as well as ectopic migration and invasion, are important characteristics of cancer cells. In order to confirm the role of CT45A1 in the metastasis of osteosarcoma, the in vivo metastatic assays revealed that the number of mice that showed metastasis following the injection of CT45A1-overexpressing osteosarcoma cells was more than the number of mice that showed metastasis following the injection of the control cells (Fig. 3A), meanwhile lung metastasis occurred more easily in groups with CT45A1 overexpression (Fig. 3B, C). Moreover, transwell and matrigel assays were performed, and we revealed that the overexpression of CT45A1 in U2OS and HOS cells significantly increased the number of cells that migrated or invaded through the membrane (Fig. 4A) but CT45A1 silencing in MG63 cells restrained these abilities (Fig. 4B). It has been reported that EMT was the key progress in metastasis of tumors and some proteins were used as markers of EMT. In western blotting, suppressed expression of E-cadherin (the marker of epithelial cell) and upregulated expression of vimentin, α-SMA, and N-cadherin (markers of mesenchymal cells) were detected (Fig. 4C) meanwhile opposite expressions of these protein markers were detected in MG63 cells with silent CT45A1 which suggested that CT45A1 could induce EMT in osteosarcoma cells (Fig. 4E). The data above suggested that CT45A1 promotes metastasis of osteosarcoma cells both in vitro and vivo through inducing EMT.

### CT45A1 promotes the progression of osteosarcoma through β-catenin

**Fig. 5:**
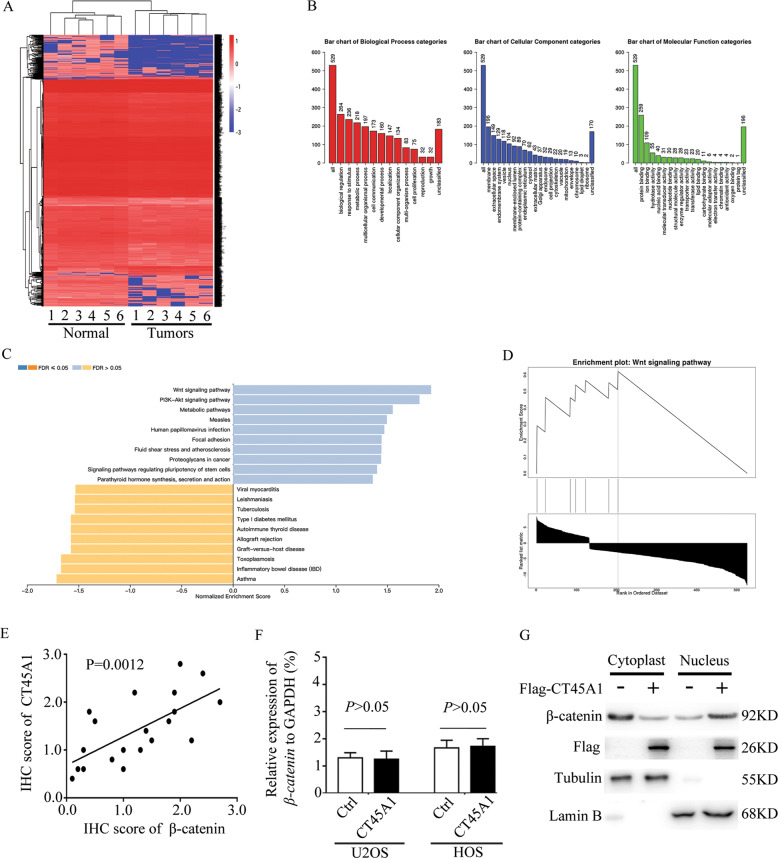
CT45A1 promotes the expression of EMT markers through β-catenin. **A** RNA-seq analysis of U2OS cells with CT45A1 overexpression. **B** GO analysis of data in (**A**). **C**, **D** KEGG analysis of data in (**A**). **E** linear correlation analysis of CT45A1 and β-catenin in osteosarcoma. **F** qRT-PCR of β-catenin in osteosarcoma cell lines with CT45A1 overexpression. **G** WB of β-catenin in cytoplast and nucleus in osteosarcoma cells with CT45A1 overexpression.

**Fig. 6:**
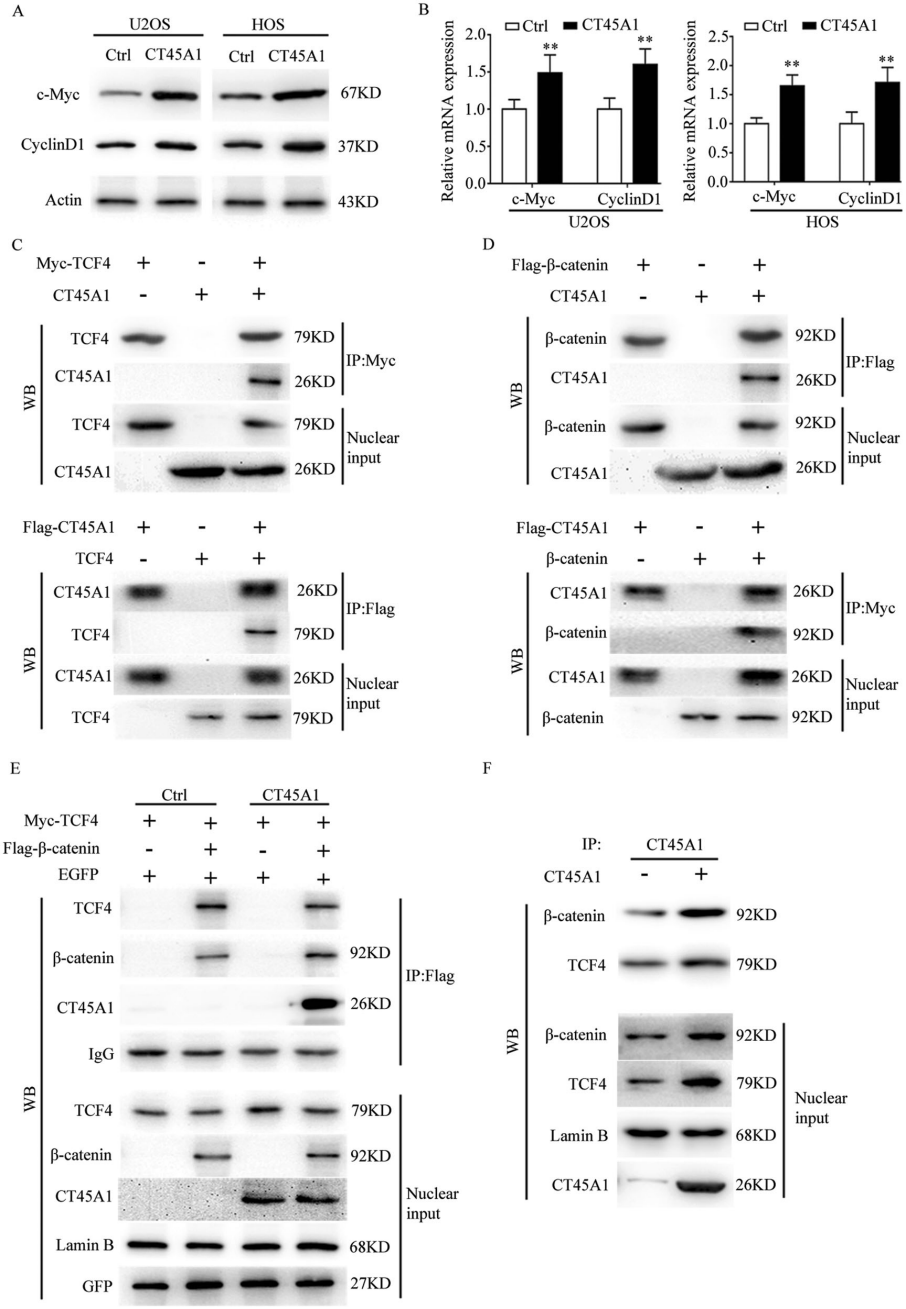
CT45A1 activates β-catenin through participating in formation of the transcriptional complex of β-catenin. **A** Western blotting for the quantification of c-Myc and CyclinD1 protein expressions in osteosarcoma cell lines. **B** qRT-PCR for the quantification of c-Myc and CyclinD1 protein expressions in osteosarcoma cell lines. **C** Co-IP analysis between CT45A1 and TCF4. **D** Co-IP analysis between CT45A1 and β-catenin. **E** Co-IP analysis between TCF4 and β-catenin under the condition of CT45A1 overexpression. **F** Co-IP analysis between CT45A1 and TCF4、β-catenin. **P < 0.01, compared to the control. The error bars indicate the means ± SDs.

With regarding to the mechanism underlying the effects of CT45A, RNA-seq analysis was conducted in U2OS cells with or without CT45A1 overexpression (Fig. 5A). Subsets of the downregulated/upregulated genes by overexpressed CT45A1 in three-replicates were identified (Fig. 5B). The GO and KEGG analysis revealed that the upregulated genes overlapped with the Wnt signaling pathway (Fig. 5C, D). To further confirm the relationship between CT45A1 and Wnt signaling pathway, linear correlation analysis was performed and we found that CT45A1 was positively related with β-catenin (Fig. 5E). It has been reported that CT45A1 was one transcription factor. In our work, CT45A1 did not change the mRNA level of β-catenin (Fig. 5F), but increased the protein level of β-catenin (Fig. 4C) as well as the expression of β-catenin in nucleus (Fig. 5G). In order to reveal the special mechanism, we first found the increased expressions of c-Myc and CyclinD1 both at protein and mRNA levels (Fig. 6A, B) which suggested that β-catenin was activated in nucleus. Based on the results, we hypothesized that CT45A1 promotes the translocation of β-catenin into the nucleus. First, Co-IP analysis was done in nuclear extract, and the results revealed that CT45A1 combined with TCF4 (Fig. 6C) or β-catenin (Fig. 6D) suggesting us that CT45A1 possibly participate in the formation of β-catenin transcription complex. Then, the Co-IP analysis was performed using anti-flag (flag-β-catenin) antibody (Fig. 6E) or anti-CT45A1 antibody (Fig. 6F) which verified that CT45A1, β-catenin and TCF4 were combined and CT45A1 could promote the combination between TCF4 and β-catenin in nucleus.

In a summary, CT45A1 could promote the translocation into nucleus of β-catenin. but further research is needed to validate the special mechanism of CT45A1 in β-catenin translocation.

## Discussion

CT45A1 is a member of the CTA family; it is correlated with multiple tumors. In this paper, we firstly revealed the function and possible network of CT45A1 in osteosarcoma metastasis. We found that CT45A1 was a risk factor in osteosarcoma because that CT45A1 overexpression suggested poorer prognosis in osteosarcoma patients and was closely with clinical pathological grade and metastasis of osteosarcoma. Based on our results, the overexpression of CT45A1 significantly promoted the proliferation and metastasis of osteosarcoma cells in vitro and in vivo. In contrast, CT45A1 silencing remarkably showed the opposite effects. According to the mechanism underlying these effects, β-catenin which was considered to play an important role in EMT during tumor progression contributed to the mesenchymal changes of osteosarcoma cells with CT45A1 overexpression by regulating the expression of EMT-related proteins. These results suggested a novel role of CT45A1 in the proliferation and metastasis of osteosarcoma cells.

The clinical treatment of osteosarcoma remains a considerable challenge because that metastasis was very likely to occur when the osteosarcoma patients were diagnosed [26]. Moreover, advanced phenotypes commonly suggested distant metastasis which is usually induced after the tumor cells showed EMT [27]. In our paper, we revealed the function of CT45A1 in the metastasis of osteosarcoma cells suggested that osteosarcoma patient with higher CT45A1 expression would show poorer prognoses because of metastasis, which also could be confirmed by the results of the overall survival analysis and IHC analysis. In clinical practice, evaluation of the prognosis of osteosarcoma patients is very important for the selection of appropriate treatment methods. Therefore, we first evaluated the role of CT45A1 in the metastasis of osteosarcoma and suggested its potential to serve as a marker for the prognosis of osteosarcoma patients.

In tumors, various transcription factors are aberrantly expressed in tumor patients with poor prognosis or therapeutic failure. According to the results of GO and KECG analysis, Wnt-β-catenin pathway was the most relevant pathway with CT45A1. β-catenin, as a typical transcription factor associated with EMT or metastasis, has been reported to play an important role in kinds of tumors [28]. Remarkably, activation of β-catenin contributed to the metastasis of osteosarcoma cells and suggested a poor prognosis of osteosarcoma as well as resistance to chemotherapy [29]. In this research, we found that CT45A1 overexpression could promote the translocation into nucleus of β-catenin and activate β-catenin pathway suggesting that CT45A1 may be a novel gene causing metastasis of osteosarcoma cell via increasing EMT-related proteins in osteosarcoma cells. These data suggested us that CT45A1 may be a potential marker for prognosis and a potential therapeutic target in osteosarcoma.

Based on the results of Fig. 5A–D, we surprisingly find that CT45A1 was not only related with Wnt-β-catenin pathway, but also P13K-Akt pathway and focal adhesion pathway. We also observed a crosstalk between these three pathways that been reported as that GSK3 could induce phosphorylation of beta-catenin and promote its degradation [30], and whether CT45A1 plays a role in the crosstalk needs future study.

In summary, CT45A1 was correlated with the prognosis and metastasis of osteosarcoma patients and acted as an oncogene in the proliferation, migration, and invasion of osteosarcoma cells which suggested that CT45A1 maybe a possible target for the osteosarcoma diagnosis and treatment.

## Supplementary information

supplementary figure 1

supplementary figure 2

supplementary figure legends
