# Supplementary figures and images for: CT45A1 promotes the metastasis of osteosarcoma cells in vitro and in vivo through β-catenin
Source: Cell Death Dis. 2021 Jun 25;12(7):650. doi: 10.1038/s41419-021-03935-x (PMC8233386; doi:10.1038/s41419-021-03935-x)

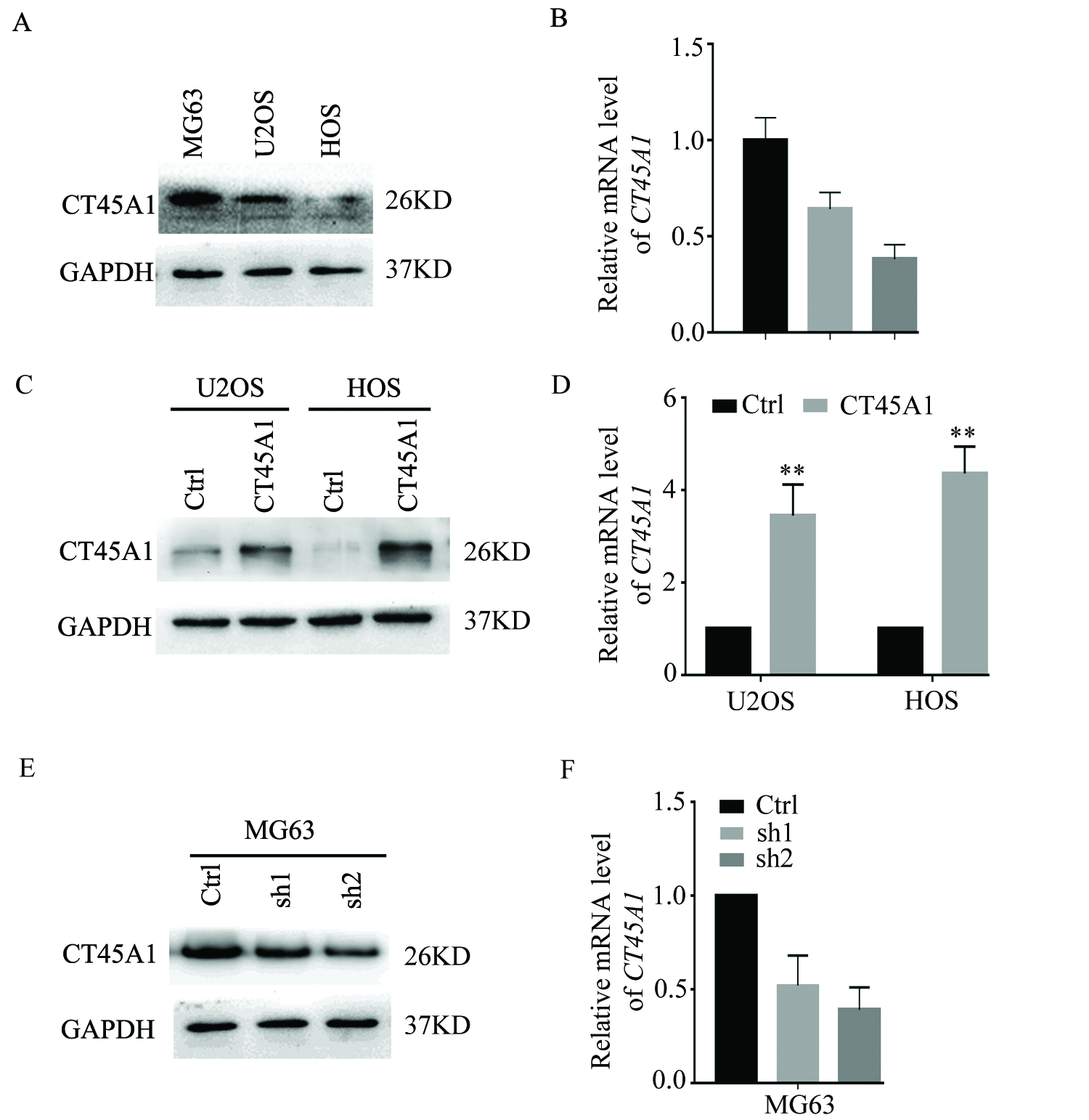

Supplement: Supplementary file 1 — supplementary figure 1 [file 41419_2021_3935_MOESM1_ESM.tif]

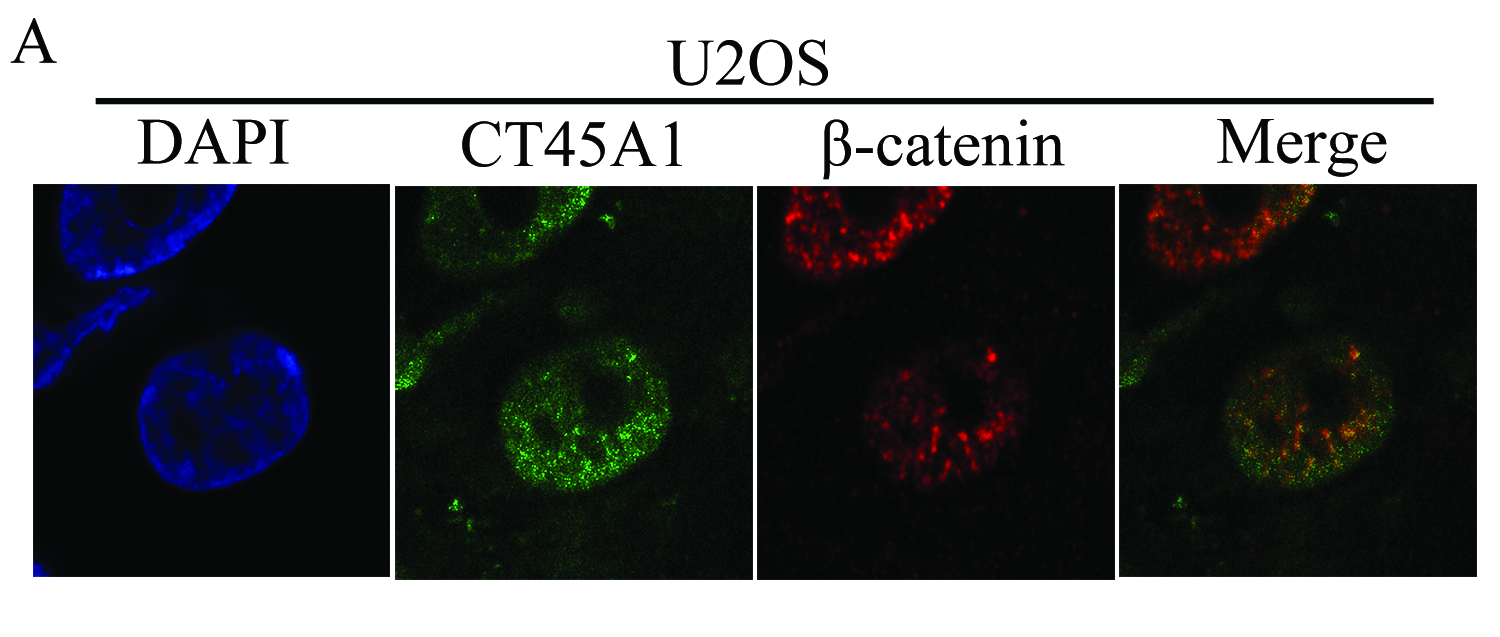

Supplement: Supplementary file 2 — supplementary figure 2 [file 41419_2021_3935_MOESM2_ESM.tif]
