# Supplementary material for: CT45A1 promotes the metastasis of osteosarcoma cells in vitro and in vivo through β-catenin
Source: Cell Death Dis. 2021 Jun 25;12(7):650. doi: 10.1038/s41419-021-03935-x (PMC8233386; doi:10.1038/s41419-021-03935-x)
Supplement: Supplementary file 3 — supplementary figure legends [file 41419_2021_3935_MOESM3_ESM.docx]

Supplemental figure 1. Expression level of CT45A1 in indicate cell lines. (A) Western blotting for the quantification of CT45A1 protein expressions in osteosarcoma cell lines. (B) qRT-PCR for the quantification of CT45A1 expressions in osteosarcoma cell lines. (C) Western blotting for the quantification of CT45A1 protein expressions in the indicated osteosarcoma cells with CT45A1 overexpression. (D) qRT-PCR for the quantification of CT45A1 expressions in the indicated osteosarcoma cells with CT45A1 overexpression. (E) Western blotting for the quantification of CT45A1 protein expressions in the indicated osteosarcoma cells with CT45A1 silencing. (F) qRT-PCR for the quantification of CT45A1 expressions in the indicated osteosarcoma cells with CT45A1 silencing. ***P*<0.01, compared to the control. The error bars indicate the means ± SDs.

Supplemental figure 2. CT45A1 and β-catenin are co-located together. (A) immunofluorescence/confocal to detect the location of CT45A1 and β-catenin in U2OS cells with CT45A1 overexpression.
